# Identification of a Novel Pathogen Using Family-Wide PCR: Initial Confirmation of COVID-19 in Thailand

**DOI:** 10.3389/fpubh.2020.555013

**Published:** 2020-10-07

**Authors:** Supaporn Wacharapluesadee, Rome Buathong, Sopon Iamsirithawon, Walairat Chaifoo, Teerada Ponpinit, Chanida Ruchisrisarod, Chanikarn Sonpee, Panticha Katasrila, Siriporn Yomrat, Siriporn Ghai, Sunee Sirivichayakul, Pilailuk Okada, Nanthawan Mekha, Opart Karnkawinpong, Sumonmal Uttayamakul, Apichart Vachiraphan, Tanarak Plipat, Thiravat Hemachudha

**Affiliations:** ^1^Thai Red Cross Emerging Infectious Diseases Health Science Centre, World Health Organization Collaborating Centre for Research and Training on Viral Zoonoses, King Chulalongkorn University, Pathumwan, Thailand; ^2^Faculty of Medicine, Chulalongkorn University, Bangkok, Thailand; ^3^Department of Disease Control, Ministry of Public Health, Nonthaburi, Thailand; ^4^Department of Medical Sciences, Ministry of Public Health, Nonthaburi, Thailand; ^5^Department of Disease Control, Bamrasnaradura Infectious Disease Institute, Ministry of Public Health, Nonthaburi, Thailand

**Keywords:** SARS-CoV-2, coronavirus, COVID-19, family-wide PCR, novel pathogen, NGS, Thailand

## Abstract

In resource-limited countries, early detection of novel pathogens is often challenging, due to financial and technical constraints. This study reports the efficacy of family-wide polymerase chain reaction (PCR) in screening, detecting, and identifying initial cases of the novel SARS-CoV-2 in Thailand. Respiratory secretions were collected from suspected individuals traveling from Wuhan, China to Thailand at the beginning of January 2020. Family-wide PCR assays yielded positive results for coronavirus in one traveler within 12 h on January 8, 2020. Nucleotide sequences (290 bp) showed 100% similarity to SARS-CoV-2. The whole genome sequence was further characterized by Next Generation Sequencing (NGS) for confirmation. Combining family-wide PCR, as a rapid screening tool, with NGS, for full genome characterization, could facilitate early detection and confirmation of a novel pathogen and enable early containment of a disease outbreak.

## Background

The accurate and timely identification of novel pathogens presents an obvious challenge in resource-limited settings, requiring expensive laboratory infrastructure and equipment along with the associated consumable supplies and reagents, and highly-trained technical staff. Timely diagnosis helps contain or prevent potential outbreaks, as in the case of the first imported Middle East Respiratory Syndrome (MERS) case in Thailand (1).

Bangkok was among the highest risk locations for the pneumonia-causing Corona Virus Disease 2019, given the large number of Chinese visitors annually. From beginning of 2020 until January 28, 2020 alone, Immigration Division 2, Thailand indicated that Thailand received 930,965 Chinese travelers, of whom 20,271 were from Wuhan (2). Total 41,000 people traveled from Wuhan to Thailand between December 1, 2019 and January 30, 2020 (2). According to the official Immigration Bureau website, Thailand received 1,054,891, 170,840, and 65,617 travelers from China in January, February, and March 2020, respectively (3).

A cluster of pneumonia of unknown origin in Wuhan, China in December 2019, presented a challenge for laboratories in resource-limited countries. Currently, the most straightforward approach to identifying unknown pathogens in humans and animals is metagenomics next generation sequencing (NGS) technology, which however has the disadvantage of being costly and time-consuming (4–6).

Family-wide polymerase chain reaction (PCR) assays have previously been used to detect various pathogens such as paramyxoviruses and MERS-CoV (7–10). Our laboratory modified and applied an existing family-wide PCR assay (11–14) to detect the novel pathogen from individuals suspected of being part of the Wuhan outbreak. We detected and successfully identified the virus on January 8, 2020. On January 10, 2020, the novel Severe Acute Respiratory Syndrome Coronavirus 2 (SARS-CoV-2) was publicly identified as the cause of the outbreak (15). In the weeks following the viral genome publication, numerous diagnostic PCR assays with specific primers and probes became available both publicly and commercially (16–18). In the interim, however, diagnostically applicable data were unavailable, and family-wide PCR was used to screen and confirm 14 COVID-19 patients in Thailand. Here, we report the process of rapidly detecting the novel virus in an imported case of COVID-19 from Wuhan to Bangkok, Thailand.

## Methods

Beginning on January 3, 2020, Thailand implemented measures for screening patients arriving at four international airports from Wuhan and stepped up surveillance at public and private hospitals. Public health nurses evaluated all arriving passengers for signs of fever (>37.5°C) and respiratory symptoms. Between January 4 and 8, 2020, specimens were collected from five individuals exhibiting fever or signs of respiratory symptoms [patients under investigation (PUIs)] identified by the Department of Disease Control (DDC), Ministry of Public Health Thailand, and sent to laboratories for diagnostic testing. Two specimen types were collected from each PUI, sputum (SP), and nasopharyngeal and throat swabs (NT) placed in viral transport media. All PUIs were transported to the Bamrasnaradura Infectious Disease Institute (BIDI) and quarantined.

All specimens collected were sent to three separate laboratories to test for 33 common respiratory pathogens using a commercial diagnostic multiplex PCR assay (Fast Track Diagnostics, Luxembourg) at BIDI, PCR analysis for SARS- and SARS-related coronaviruses (CoVs) at the Department of Medical Sciences laboratory, Ministry of Public Health (DMSc), and to the Thai Red Cross Emerging Infectious Diseases Health Science Centre (TRC-EID) laboratory, where family-wide PCR testing was performed for influenzas and coronaviruses using established protocols (11–14) (Table 1).

**Table 1:** PCR results of 33 known respiratory pathogen and family-wide PCR detection for 5 PUIs identified.

**Specimen Type**	**Known pathogen detected**	**3 CoV family-wide PCRs (Sequencing result)**	**Pan-Influenza A PCR (Sequencing result)**
**PATIENT #1**			
Throat Swab	Influenza A virus	Negative	Positive (Influenza A virus)
	*Moraxella catarrhalis*		
	*Haemophilus influenzae*		
Suction	Influenza A virus	Negative	Positive (Influenza A virus)
	*Moraxella catarrhalis*		
	*Haemophilus influenzae*		
**PATIENT #2**			
Nasopharyngeal and throat swabs	Respiratory Syncytial Virus A/B	Negative	Negative
	*Staphylococcus aureus*		
	*Klebsiella pneumoniae*		
Sputum	Respiratory Syncytial Virus A/B Influenza C virus	Negative	Negative
	*Staphylococcus aureus*		
	*Klebsiella pneumonia*		
	*Haemophilus influenzae*		
**PATIENT #3**			
Nasopharyngeal and throat swabs	*Haemophilus influenzae*	Negative	Negative
Sputum	*Haemophilus influenzae*	Negative	Negative
**PATIENT #4**			
Nasopharyngeal and throat swabs	Coronavirus OC43	Positive—Protocol W	Negative
	*Haemophilus influenzae*	Positive—Protocol Q	
		Positive—Protocol C (Coronavirus OC43)	
**PATIENT #5**			
Nasopharyngeal and throat swabs	*Staphylococcus aureus*	Positive—Protocol Q	Negative
	*Haemophilus influenzae*	Positive—Protocol C (SARS-CoV-2)	
Sputum	*Staphylococcus aureus*	Positive—Protocol Q	Negative
	*Haemophilus influenzae*	Positive—Protocol C (SARS-CoV-2)	

The family-wide approach uses three pan-CoV nested Reverse Transcription PCR (RT-PCR) assays labeled W, Q, and C to amplify different regions of the RNA-dependent RNA polymerase gene (RdRp) (8–10). All three PCR assays were run separately using the same reagents for all patient samples. The 5 μL RNA template was added to 45 μL One Step RT-PCR master mix reagents (Qiagen) containing 10 μL of 5 × buffer, 2 μL of dNTP (10 mM), 2 μL of Enzyme mix, and 1.5 μL of each primer (10 μL). Thermal cycling was performed according to each published protocol (12–14).

All PCR positive amplicons were further characterized by direct sequencing for confirmation. Additionally, whole genome sequencing (WGS) using NGS technology was performed on respiratory specimens from patients whose family-wide CoV PCR was positive for SARS-CoV-2. WGS was performed using TruSeq RNA library preparation and an Illumina MiSeq 3000 sequencer, according to the manufacturer's instructions with subsequent *de novo* assembly and reference mapping analysis. WGS was also performed at DMSc (19).

Once specific primer was available, real-time PCR (qPCR) assay targeting the receptor-binding domain of the spike gene using SYBR-Green One Step RT-PCR was later performed according to the published protocol (17) on respiratory tract specimens from patients whose SARS-CoV-2 testing was previously positive by family-wide PCR.

## Results

Detailed results of the 5 PUIs are shown in Table 1, where Patients #1–4 were negative for SARS-CoV-2, and positive for various different pathogens. Patient #5, whose specimen was collected on January 8, 2020, was found positive for novel coronavirus, which was later confirmed as SARS-CoV-2. The patient's NT and SP specimens were positive for 2 pan-CoV PCR protocols (Q and C), and negative for protocol W and all other viruses. Upon further investigation, sequence mismatch (up to 7 in 20 nucleotides) between the primers in protocol W and SARS-CoV-2 (GenBank accession no. NC_045512.2) was observed, which seemed to reduce the efficacy of the assay for SARS-CoV-2.

Direct sequencing was conducted from these PCR amplicons and the nucleotide sequences from both Q and C protocols [290 and 182 base pairs (bp), respectively] which were both positive for SARS-like-CoV, albeit different regions of the RdRp gene. A GenBank BLAST search performed on January 9, 2020 showed the sequences were best-matched (83.1% and 93.4%, respectively) to bat SARS-like-CoV isolate 4231 (GenBank accession no. KY417146.1). Repeat analysis on January 11, 2020, 1 day following the online publication of the novel 2019 CoV (i.e., SARS-CoV-2, GenBank accession no. MN908947.3) genome (15), showed a complete match (100% concordance) for both fragments (the 290 nucleotide sequence from sputum is published in GenBank; Accession no. MN970003).

WGS of this patient's NT specimen using NGS, (GenBank Accession no. MT447155), yielded a 29,805 nucleotide sequence identified as SARS-CoV-2, based on a 100% (29,805/29,805 bp) match to the virus identified in patients at the beginning of the outbreak in Wuhan (GISAID accession no. PI_ISL_402124) (17).

Upper and lower respiratory tract specimens from the index patient were retested over 7 subsequent days, with positive results in most specimens (Table 2). Testing was discontinued after yielding negative results on 2 consecutive days and the patient returned to China.

**Table 2:** PCR results of the specimens from Patient #5 collected during January 8 to January 18, 2020.

**Collection date**	**Specimen #**	**Specimen Type**	**CoV PCR Protocol Q**	**CoV PCR Protocol C**	**qPCR (threshold cycle)**
8 January 2020	200040-NT	NT	100% identity to SARS-CoV-2	100% identity to SARS-CoV-2	20.166
	200040-SP	Sputum	100% identity to SARS-CoV-2	100% identity to SARS-CoV-2	NA^*^
9 January 2020	200049-NT	NT	Not Detected	Not Detected	30.847
	200049-SP	Sputum	Not Detected	Not Detected	29.628
12 January 2020	200107-NT	NT	100% identity to SARS-CoV-2	100% identity to SARS-CoV-2	22.949
	200107-SP	Sputum	100% identity to SARS-CoV-2	100% identity to SARS-CoV-2	NA^*^
14 January 2020	200129-NT1	NT	Not Detected	Not Detected	32.032
	200129-SP1	Sputum	100% identity to SARS-CoV-2	100% identity to SARS-CoV-2	21.929
	200129-NT2	NT	Not Detected	Not Detected	32.032
	200129-SP2	Sputum	100% identity to SARS-CoV-2	100% identity to SARS-CoV-2	23.516
15 January 2020	200136-NT	NT	Not Detected	Not Detected	31.47
	200136-SP	Sputum	100% identity to SARS-CoV-2	100% identity to SARS-CoV-2	25.66
16 January 2020	200154-SP	Sputum	100% identity to SARS-CoV-2	100% identity to SARS-CoV-2	29.2
17 January 2020	200182-NT	NT	Not detected	Not detected	Not Detected
18 January 2020	200209-NT	NT	Not Detected	Not Detected	Not Detected

* *NA, specimen was not available for testing. NT, Nasopharyngeal and throat swabs*.

To determine the sensitivity and specificity of family-wide PCR, the specimens from the index patient were re-tested using qPCR (17). The qPCR analysis gave concordant results to family-wide PCRs (both Q and C protocols) when the qPCR threshold cycle (ct) was lower than 29.6 (Table 2). The qPCR provided better sensitivity than family-wide PCR in specimens with lower viral load. Specimens from Patients 1 to 4 were all negative when re-tested with qPCR using specific primer for SARS-CoV-2.

## Discussion

The magnitude of an outbreak can substantially be reduced by rapid and early identification of cases (20). This report shows the value of family-wide PCR when applied to detect unknown viruses, which led to the rapid identification of SARS-CoV-2 in Thailand. This report focuses on diagnoses of the initial patients and is not a comprehensive examination of the clinical, epidemic, and laboratory review of early COVID-19 cases in Thailand. SARS-CoV-2 was detected and identified in Thailand 1 day before China confirmed the cause of the Wuhan epidemic (15). The suspected importation of the novel coronavirus was unofficially communicated to the World Health Organization in-country office. All positive PCR amplicons were further sequenced for confirmation, to identify the viral species and verify that it was not a false positive from non-specific binding or contamination. The efficient detection of this pathogen is the result of coordinated public health efforts with collaborations between several Thai laboratories, allowing rapid results and proficient outbreak containment measures. Despite being the first country to confirm a case of COVID-19 outside of China at the beginning of January, <100 confirmed COVID-19 cases were registered in Thailand for more than 2 months, until the marked increase in new cases in mid-March (21).

The short PCR amplicon from family-wide PCR generates less sequence information when compared to the sequence information from NGS. This study showed that the two family-wide PCR protocols (protocol Q and C) exhibited similar sensitivities despite having different amplicon fragment lengths (328 and 228 bp, respectively), which could potentially affect diagnostic accuracy, as sequence length correlates with specificity. Our data from Patient #5 (Table 2) suggests that the sensitivity of family-wide PCR is lower than qPCR, which may result in false negative results if the viral load in the specimen is low. The qPCR (commercially available or published in-house protocols) has been used at our laboratory for routine diagnosis of COVID-19 once specific primers and probes were available, which provides more rapid results. A shortcoming to family-wide PCR is the need for guesswork involved in choosing which viral family to amplify. CoV and influenza viral families were chosen for first tier testing during this investigation as these are common respiratory pathogens causing pneumonia.

Despite the shortcomings, family-wide PCR is convenient and sensitive as a screening assay for an unknown pathogen. Moreover, the method has several advantages, notably being more cost effective (about 100 × less costly), more efficient (2–4 × faster than NGS), and simpler, as no special training is required for laboratory personnel beyond routine PCR. Previously, this laboratory approach was successfully used to identify the first MERS case in Saudi Arabia from viral culture specimens (8). Family-wide PCR has also been used for pathogen detection in wildlife under USAID's PREDICT project, Emerging Pandemic Threats (EPT) program since 2010 (9, 10). It is conventionally recommended as a supplemental method for the detection of novel viruses rather than the main detection assay, as it is less sensitive than qPCR (11).

## Conclusion

Our results demonstrate the practical applications of family-wide PCR as the initial detection assay for novel viruses of extreme pathogenicity in limited-resource setting when specific primers are not available, generating results within 12 h. The short turnaround time is much preferred to NGS which can be both time-consuming and costly. The efficient containment of an outbreak of any novel pathogen requires rapid identification of the novel pathogen, and family-wide PCR was well suited to this purpose.

## Data Availability Statement

The datasets presented in this study can be found in online repositories. The names of the repository/repositories and accession number(s) can be found here: https://www.ncbi.nlm.nih.gov/nuccore/MN970003 [GenBank Accession No. MN970003].

## Ethics Statement

Ethical review and approval and written informed consent for participation was not required for clinical specimens collected for outbreak investigation purposes in accordance with the national legislation and institutional requirements.

## Author Contributions

SW, RB, SI, WC, SS, PO, NM, OK, SU, AV, TPl, and TH involved in the study design. TPo, CR, CS, PK, and SY conducted the diagnostic analyses. SG drafted the manuscript. SW and SG critically reviewed the manuscript. All authors contributed to the article and approved the submitted version.

## Conflict of Interest

The authors declare that the research was conducted in the absence of any commercial or financial relationships that could be construed as a potential conflict of interest.
